# MarketTrust: blockchain-based trust evaluation model for SIoT-based smart marketplaces

**DOI:** 10.1038/s41598-023-38078-w

**Published:** 2023-07-18

**Authors:** Rabia Latif, Bello Musa Yakubu, Tanzila Saba

**Affiliations:** 1 Artificial Intelligence and Data Analytics Laboratory (AIDA), College of Computer and Information Sciences (CCIS), Prince Sultan University, 11586 Riyadh, Saudi Arabia; 2 Department of Mathematics and Computer Science, Faculty of Science, Chulalongkorn University, Bangkok, Thailand

**Keywords:** Energy science and technology, Engineering, Mathematics and computing

## Abstract

Due to the significance of trust in Social Internet of Things (SIoT)-based smart marketplaces, several research have focused on trust-related challenges. Trust is necessary for a smooth connection, secure systems, and dependable services during trade operations. Recent SIoT-based trust assessment approaches attempt to solve smart marketplace trust evaluation difficulties by using a variety of direct and indirect trust evaluation techniques and other local trust rating procedures. Nevertheless, these methodologies render trust assessment very sensitive to seller dishonesty, and a dishonest seller may influence local trust scores and at the same time pose a significant trust related threats in the system. In this article, a MarketTrust model is introduced, which is a blockchain-based method for assessing trust in an IoT-based smart marketplace. It has three parts: familiarity, personal interactions, and public perception. A conceptual model, assessment technique, and a global trust evaluation system for merging the three components of a trust value are presented and discussed. Several experiments were conducted to assess the model's security, viability, and efficacy. According to results, the MarketTrust model scored a 21.99% higher trust score and a 47.698% lower average latency than both benchmark models. Therefore, this illustrates that using the proposed framework, a potential buyer can efficiently choose a competent and trustworthy resource seller in a smart marketplace and significantly reduce malicious behavior.

## Introduction

The Internet of Things (IoT) links several electronic gadgets to the Internet, these include Radio Frequency Identification cell phones, smart home appliances, and smart automobiles^1^. In a proposal dating back 30 years, scientists proposed a worldwide web of connections that would bring together the real world, the virtual world, and actual people^2^. In the last ten years, Semantic Web and others have expanded upon this concept. Through IoT, we're creating a Cyber-Real-Social System that connects Cyber-Social Webs to real-world objects^3^. IoT will monitor global human existence using billions of detecting and actuating gadgets. Observation data are aggregated, processed, and analyzed to describe real-world occurrences. The combination of cyber and social data inside a service has the potential to reveal untapped operational efficiencies and provide a continuous virtuous cycle between consumer demands and product replies^4^. An IoT design that prioritizes people and their needs above technology and hardware has been developed: the Cyber-Physical-Social System^5^.

Prior until now, most IoT-related research articles concentrated on Radio frequency identification and Wireless Sensor Networks to enable communications and interactions between physical things and the Internet^6^. Human-centric Internet of Things ecosystems, in which people support services and applications, are gaining prominence^7^. When creating real-world IoT applications, social phenomena and crowd intelligence are widely used. To exemplify the concept of the Cyber-Real-Social System, the so-called Social Internet of Things (SIoT) has been proposed^3,7^. The SIoT combines physical objects, cyber components, and humans, hence creating new threats, privacy, and security issues. Managing risk and protecting the SIoT are more complicated than the traditional trinity of availability, confidentiality, and integrity^8^. When making choices, trust helps people and services overcome ambiguity and risk. One important segment of social services offered by SIoT nowadays is smart marketplaces^9^. It enables individuals to exchange resource products for their daily leaving.

Due to the importance of trust in SIoT, several studies have focused on trust-related challenges in peer-to-peer networks, wireless sensor networks, social networks, and the IoT^10^. Trust is a complex concept that is influenced by both individuals and their surroundings. In SIoT-based smart marketplaces, a buyer decides whether to risk a malicious seller based on a psychological evaluation. Smart marketplaces aim to make decisions autonomously without human intervention; hence, trust is essential for a seamless connection, secure systems, and reliable services. A trust platform may reduce unplanned risks and boost predictability, enabling SIoT platforms and services such as smart markets to operate in a regulated manner and preventing unanticipated events and disruption of services^11^.

Researchers have devised trust building, distribution, updating, and maintenance mechanisms^12^. Some research demonstrates smart marketplace-based trust assessment algorithms based on direct trust, extracted by observing seller characteristics or behavior. This data is used to describe a seller's trust-related characteristics, known as Trustworthiness Attributes^8,13^. These characteristics are used to generate an overall trustworthiness grade. Other ways measure trust based on a buyer's prior interactions with a seller, which is indirect trust assessment^14,15^. This involves analyzing a seller's perspectives after each interaction and propagating the resulting comments and ideas by a buyer. In nutshell, indirect trust involves combining third-party information to assess a seller's reputation. In another scenario, a buyer may connect with co-located sellers in smart marketplaces to select trade tasks and resources. Either high-quality or low-quality trade services may be provided by sellers^16^. Defamation, voting, self-promotion, and whitewashing attacks may be employed by malicious sellers in this regard. Consequently, a trustworthy ecosystem must be based on an efficient mechanism for evaluating trust^17^.

Recent SIoT-based trust assessment methods^9,18^ try to address smart market/service provisioning trust evaluation issues. However, neither social systems nor SIoT service computing settings are considered^19^. Similarly, the existing trust assessment methodologies for smart markets based on SIoT do not permit in-depth social interactions between entities^20^. Some mechanisms evaluate the dependability of sellers in a dynamic manner. In other words, they examine past interactions to identify malicious attacks and selfishness. As a result, these systems of trust assessment are divided into decentralized and centralized components^2^.

A central authority manages the credibility of centralized systems. Therefore, this centralized method has a single point of failure and bottleneck issues^21^. The decentralized trust management architecture is presented to address issues in centralized systems, in which a buyer is required to calculate and maintain the local trust scores of other sellers with whom it has interacted^22^. A buyer may consider the trust recommendations of others when calculating local trust. Decentralized design offers numerous advantages, but in practice it is inefficient because a dishonest seller can manipulate local trust ratings. This makes trust evaluation susceptible to seller deceit^21,22^. Second, a buyer may lack the computational capacity to utilize the trust computation of existing models. Consequently, determining the reliability of smart market participants is a pressing concern. This research aims to develop a trustworthy distributed trust assessment system for smart marketplaces. However, blockchain technology can provide a remedy^21,22^.

By linking hashes to transactions, blockchain provides an immutable audit trail of sensor observations in the SIoT^23^. Transactions are organized into blocks that are connected to prior blocks in the chain via cryptographic hash functions, making it nearly impossible to modify previously recorded blocks. Before adding them, Blockchain may validate SIoT transactions and blocks using public key cryptography^24^. Once blocks are mined into the blockchain, we guarantee the integrity and immutability of inter-node transactions^21,22^.

This paper presents MarketTrust, a blockchain-based technique for assessing trust in an SIoT-based smart marketplace. It has three parts: familiarity, personal interactions, and public perception. A conceptual model, assessment methodologies, and a global trust evaluation system for integrating the three components into a trust value are presented and discussed.

Our contributions are in the following:

- Presented is a blockchain-based trust assessment methodology for SIoT-based smart marketplaces that enables the measuring of trust based on social relationships between trade partners. Individuals (buyers) typically base their assessment of a seller's trustworthiness on three key pillars: familiarity with the seller (Familiarity), prior interactions with the seller (Personal Interactions), and public perception of the seller (Public Perception). This makes the trust model highly adaptable to dynamic scenarios.
- The approach utilizes blockchain technology to choose resource products based on sellers' levels of familiarity, personal interaction, and public opinion. Consequently, although the system assures secrecy, anonymity, and a high degree of trust computation, it also assists buyers in selecting the most qualified seller and prevents dishonest parties from appearing to be trustworthy.
- Several experiments were conducted in the study to test the security, viability, and effectiveness of the proposed model, indicating that using the model, a prospective buyer can effectively select a competent and honest resource seller in the smart marketplace and significantly reduce malicious behavior.

The remaining sections of the paper are organized as follows. The second section describes the associated work. In Section "System model", the system model is presented. In Section "Proposed trust evaluation model", the proposed model for evaluating trust is described. The security analysis is included in Section "Security analysis". The sixth section details performance analysis. Section “Conclusions” finally concludes the paper.

## Related works

Several trust models that attempt to simulate the way the human brain calculates the value of a trust take into account reputation, recommendation, and observation, among other factors^7,25^. In most cases, they assess trust based on social network communications between entities, resulting in a decentralized, activity- or encounter-based form of computation. Inability to account for subjective aspects of trust in addition to objective ones is the method's flaw^2,26^. Using reputation schemes can benefit P2P, MANET, and WSN-based systems, such as a smart marketplace^27^. Taking into account buyers reviews and the overall reputation of the seller are examples of how trust-based smart marketplaces achieve this^24^. Several properties, including trustworthiness, connection factors, and transaction factors, are derived from feedbacks and incorporated into the SIoT's trust management scheme, as described by the authors in^9,25^.

Numerous trust models calculate a trust value in order to determine whether or not to conduct business with a particular seller based on a variety of features and related evaluation methods^7,18^. A subset of attributes should be selected with care, considering the buyer's predisposition and external variables, to maximize perceived dependability. As an illustration, the authors of^28–30^ used several attribute parameters to determine if a smart marketplace seller is dishonest. In^7,16,31^, the authors investigate the various trust models employed in smart marketplaces, as well as the numerous threats and vulnerabilities that could compromise their capacity to conduct secure trade operations. One disadvantage of these methods is that they do not demonstrate the subjectivity of trust by combining data in any way. Uniquely, they employ an improved Bayesian model using a weighting system that is specific to each kind of data collected by direct observation.

As previously stated, current schemes monitor entity interactions to identify attacks and self-serving behavior. They determine which entities within a smart marketplace network can be trusted in real time. Regarding the centralized mechanism for evaluating trust, one entity is primarily responsible for managing trust. Thus, the existing systems were plagued by the single point of failure and bottleneck issues, in addition to the numerous disadvantages previously mentioned^32,33^. Data security and privacy concerns arise in decentralized schemes where the roles of trading entities are fluid^24,34^. Similarly, trust ratings in a specific area can be altered relatively easily. This leaves trust evaluation vulnerable to attack from malicious actors. To address these concerns, blockchain-based trust evaluation architectures were proposed^24,32^.

In blockchain-based architectures, the local trust scores of other entities with which an entity has interacted must be computed and stored^21,35^. For a more precise evaluation of local credibility, a buyer may rely on the recommendation of others. The security, verifiability, and immutability of data transmissions and data anonymization have been enhanced by blockchain technology^36,37^. Furthermore, the technology offers a secure, decentralized database. Even though the benefits of current blockchain-based decentralized architectures are evident, it is also essential to recognize their drawbacks. Models such as^38,39^ accomplish this by having an entity (seller) precompile trust value based on the recommendations of other network entities (buyers). These methods reduce costs by assuming an entity (buyer) will act as a middleman, but they cannot be relied upon if they only utilize data from a single smart marketplace^24^. Instead, models^40,41^ utilize direct indicators of reliability, such as past behavior and the opinions of the model's entities (buyers). The studies accounted for loyalty, candor, and cooperation depending on the nature of their interpersonal relationship. Each buyer is required to maintain a data retrieval table containing their trust information. This information contains not only their own trust history, but also the trust histories and recommendations of other buyers.

As seen in^42–44^, methods for evaluating the trustworthiness of blockchain systems frequently rely on clustering (grouping/multi-agent). These methods can facilitate trade between entities with high levels of trust, thereby eliminating the possibility of dealing with dishonest sellers in smart marketplace context. These techniques frequently result in risky double-spending and require substantial computational resources^45–47^. When clusters are formed based on the similarity of their members' profiles, uniformity is not guaranteed^41^. This phenomenon is referred to as association challenges in reference to cluster recommendation^48,49^.

Consequently, the current trading methods pose potential evaluation and security risks. Modern methods are susceptible to threats like defamation, voting, and others since entities may engage in dishonest behavior to advance their careers or tarnish others' reputations. Similarly, the trust score computation mechanisms of the smart market may incur delays or additional costs. As a result, determining the dependability of smart market participants and the overall safety of the trading cycle are pressing issues. Thus, there is a need to develop a blockchain-based distributed trust assessment system for effective trading in smart marketplaces. Table 1 presents the most recent and relevant literatures based on their techniques, objectives, and limitations.

**Table 1:** Summary of the related works.

Techniques	Objectives	Limitations
Social network communications between entities^7,25^	To simulate the way the human brain performs trust computations using several factors	Inability to account for subjective aspects of trust in addition to objective ones
Selected attributes based on the buyer's predisposition and external variables^7,18^	To maximize perceived dependability	Single point of failure and bottleneck issues
Bayesian model^7,16,31^	To research smart marketplace trust models and the dangers and vulnerabilities that might impair their ability to execute safe trading operations	Does not demonstrate the subjectivity of trust by combining data in any way
Blockchain^38,39^	To compute trust of entity (seller) based on the recommendations of other network entities (buyers)	Techniques cannot be relied upon if they only utilize data from a single smart marketplace
Blockchain^40,41^	To provide trust computations based on direct indicators of reliability	The studies can be affected with high computation costs
Blockchain and grouping/multi-agent techniques^42–44^	To evaluate the trustworthiness of trading parties using clustering	Techniques can be affected with double-spending and require substantial computational resources Uniformity cannot be guaranteed clusters are used

## System model

**Figure 1:**
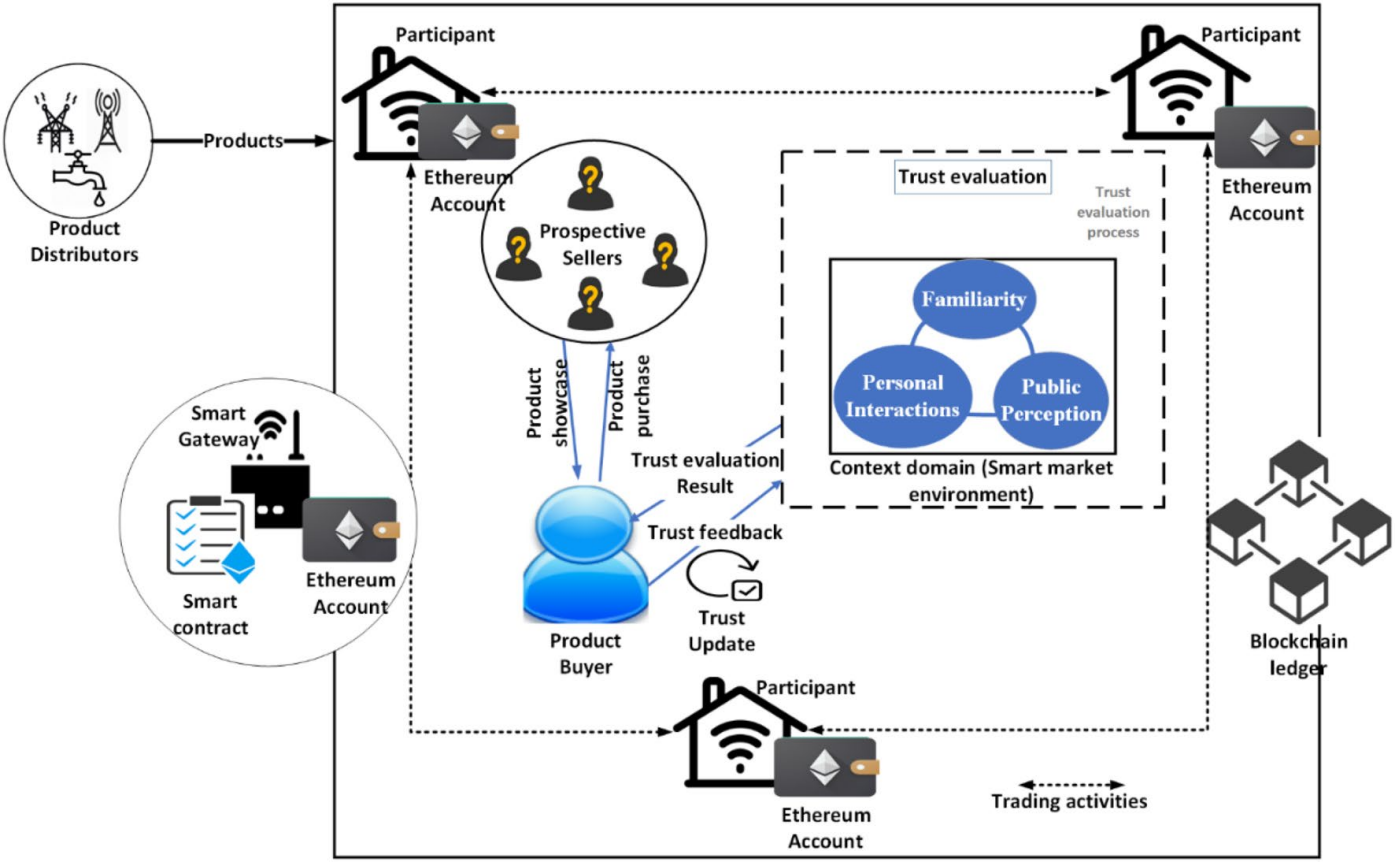
Proposed MarketTrust model.

The proposed model entails the creation of a smart marketplace comprised of many smart homes. The smart marketplace has a tamper-resistant gateway known as the Smart Gateway (SG). Every participant in the model is equipped with a sensor-enabled smart gateway that connects to the SG in the smart marketplace. As shown in Fig. 1, every member of the smart marketplace may trade product resources as a seller or a buyer. Providers of energy and telecommunications services to smart homes are examples of the types of businesses represented by the Resource Distributor (RD). Since Ethereum transactions are substantially faster than bitcoin transactions, the technique is based on a semi-centralized private Ethereum blockchain. Each connected home has access to the whole transaction history stored in the distributed ledger and has the computing prowess to participate. Additionally, each member has a unique Ethereum Account (EA) and has direct access to the network's blockchain Smart Contracts.

### Trust modelling

As per social science context, individuals (trustors) frequently base their trust assessment on three primary foundations: (i) understandings on a seller (trustee) (Familiarity); (ii) past transactions with a seller (personal interactions); and (iii) public opinion on a seller (public perception). Consequently, this study assumes that the above-mentioned socially cognitive process is relevant to the SIoT setting.

Familiarity influences a trustor's perception of a trustee's trustworthiness in a direct manner. Observing the trustee and his or her environment is a means of gaining familiarity. Environmental dangers, vulnerabilities, and risks are less revealing than the trustee's and grantor's trustworthiness.

Personal interactions and public perception are acquired social traits through prior SIoT contacts. Personal interactions are the basis for a trustor's assessment of a trustee's trustworthiness based on previous interactions. Due to the lack of information, personal perception and interactions are more indicative of the trustor's propensity than the trustee's credibility and the context.

Incorporating all past interactions with a trustee, public perception reflects a global perspective. Anyone may determine if the trustee and surrounding environment are trustworthy, but they cannot determine whether the trustor is likely to be trustworthy. In SIoT situations involving number of participants, there may be no previous interactions, resulting in no Personal interactions. Public perception is a crucial trust indicator, especially when the trustor and trustee have never interacted.

Due to the contagious nature of trust, public opinion is assessed while being evaluated. Each participant (trustor) who has had past interactions with a given participant (trustee) has views; a reputation/recommendation model allows these opinions/recommendations to be shared with others. As a trust indicator, participants may use views. This increases network integrity. By fusing the three trust sources and incorporating blockchain, the proposed trust assessment model consolidates computational trust so that it can be utilized in the majority of SIoT scenarios with high accuracy and integrity.

In this research, the trust model connection is context-dependent since the result of product selection differs according to the smart market environment. Consequently, context encompasses both the product kind and the specific setting in which the seller will supply the goods. The items that the selected seller should deliver are contained in the buyer-requested list of products $P=\{{\mathcalligra{p}}_{1},{\mathcalligra{p}}_{2}\dots , {\mathcalligra{p}}_{n}\}$. Apart from the general description, resource product ${\mathcalligra{p}}_{y}$ will contain specific characteristics or requirements that are essential for the product to succeed and meet the buyer's objectives. If the consumer chooses and purchases the product successfully, the product result PR will accomplish the aim $G=1$, otherwise, $G=0$.

This research will use trust as a quantifiable indicator to describe the degree to which a buyer (with aim $G=1$) trusts a seller $y$ of a particular product ${\mathcalligra{p}}_{y}$ when purchasing the product in context C. Our trust model in SIoT aims to assist buyers in evaluating a potential seller's trustworthiness for a particular product transaction. Therefore, when determining a seller's reliability, the buyer must consult the above three primary trust assessment sources. Table 2 provides brief descriptions of some basic symbols and abbreviations used in this model.

**Table 2:** Notations.

Symbols	Descriptions	Symbols	Descriptions	Symbols	Descriptions
SG	Smart Gateway	${\mathcalligra{T}}_{{\mathcalligra{p}}_{y}}$	Number of profitable trades accomplished from ${\mathcal{N}}_{avl}^{x}$	$\vartheta$	Parameter specifying the least decay value of personal interaction
RD	Resource Distributor	$\eta$	Success rate	$\mu$	Decay rate
EA	Ethereum Account	${\mathcalligra{w}}$	Willingness	${PI}_{y}$	Personal interaction
$P$	List of products	${\mu }_{{\mathcalligra{p}}_{y}}$	Number resource product requested	$\gamma$	Threshold parameter
${\mathcalligra{p}}_{y}$	Resource product	$\varphi$	Number of times the seller $y$ has engaged in resource product trade	$\alpha$	Damping factor
PR	Product result	$T$	Time taken to complete the profitable trades	${{\mathcal{Q}}}_{+ve}$, ${{\mathcal{Q}}}_{-ve}$	Positive public and negative public opinions respectively
$G$	Aim	$\delta$	Integrity	$n$	Total number of participants in the network
$TS$	Trust score	${\mathcalligra{C}}_{{\mathcalligra{p}}_{y}}$	The trading tasks canceled from ${\mathcal{N}}_{avl}^{y}$	${\mathcal{Q}}\left(y\right)$	The public opinion on the participant $y$
$x$,$y$	Buyer and seller respectively	${\mathcal{F}}_{y}$	Familiarity value	${\mathcalligra{r}}_{+ve}\left(x\right)$, ${\mathcalligra{r}}_{-ve}\left(x\right)$	The respective summation of all positive and negative public opinion values $x$ is currently sharing
${\mathcal{R}}_{\mathcalligra{t}}$	Response time	*A, B, C*	Weights	${min}_{{\mathcal{Q}}}$	The minimal value of public opinion
${\mathcalligra{d}}_{\mathcalligra{t}}$	Time of supply	${\sigma }_{\mathcalligra{t}}$	personal interaction at a time $\mathcalligra{t}$	${P}_{select}^{y}$	Resource product select value
${\mathcalligra{r}}_{\mathcalligra{t}}$	Time of demand	$\tau$	The highest increase value of the personal interaction	${T}_{{\mathcal{F}}_{y}}$, ${T}_{{PI}_{y}}$, and ${T}_{{\mathcal{Q}}\left(y\right)}$	The levels of sellers' familiarity, personal interaction, and public opinion, respectively
${\mathcal{N}}_{adv}^{y}$	Frequency at which a given resource product was demanded to be advertised over a period	${max}_{\sigma }$	The highest value of personal interaction	$PoA$	Proof-of-Authority
${\mathcal{N}}_{avl}^{y}$	Frequency at which the resource product was obtainable	${min}_{\sigma }$	The minimum value of personal interaction	$PoW$	Proof-of-Work
${\mathcal{V}}_{{\mathcalligra{p}}_{y}}$	Resource product availability	${\mathcalligra{D}}_{\mathcalligra{t}-1}$	Decay parameter,	$EVM$	Ethereum Virtual Machine

### Adversary model

This section describes our adversary's characteristics as shown below:

1. An adversary may remain a passive spectator and participate in dishonest behavior to get personal gain.
2. Internal enrichment and external exploitation are the two most often employed strategies by adversaries when conducting trust-related attacks.
  - The adversary use deception to increase its credibility internally via enrichment. Self-promotional attacks, whitewashing and opportunistic service are just a few examples.
  - The adversary is shown as purposefully influencing others' credibility to gain or lose a competitive advantage via external exploitation. Defamation, voting, and discriminatory attacks are three instances of this kind of activity.
3. The smart gateway is tamper-proof and consequently unbreakable.
4. The adversary is expected not to compromise the blockchain.

Notably, this solution does not address the occurrence in which 3 and 4 are compromised. Therefore, this circumstance is the important restriction of the model that we wish to discourse in our feature work.

## Proposed trust evaluation model

The proposed model is derived from the amalgamation the three basic trust evaluation sources using smart contract and blockchain technologies. The following subsections presents the formation and working principle of the proposed model.

### Global trust modeling

The proposed trust model aims to record the seller's trust for every trading task. Given a collection of all types of resource products $P=\{{\mathcalligra{p}}_{1},{\mathcalligra{p}}_{2}\dots , {\mathcalligra{p}}_{n}\}$, where ${\mathcalligra{p}}_{y}$ is a given resource product in $P$, the trust can be calculated using sellers' familiarity value, personal interaction value, and the public opinion value recorded on a seller during trading task execution. Consequently, the trust score $TS$ for a particular vendor is calculated as follows:

1 $${TS}_{y}=A\cdot {\mathcal{F}}_{y}+B\cdot {PI}_{y}+C\cdot {\mathcal{Q}}\left(y\right)$$

where A, B, and C are the equation's corresponding weights. Where $0\le A,B,C\le 1$ and $A+B+C=1$ are the weighting factors that determine how important each trust metric is. The trustor decides on these weights in order to provide the highest possible level of accuracy in the trust evaluation and to strengthen the protocol's resistance to the several attacks that have been uncovered. Moreover, Algorithm 1 provides a summary of the global trust computation operations.

### Familiarity modelling

The proposed model sets criteria for a seller's eligibility to participate as a resource seller in the smart marketplace by evaluating whether the seller provided the resource product in a healthy state and as anticipated. In other words, the buyer $x$ selects a seller $y$ in whom it has faith to complete the transaction. Consequently, to achieve a trading task, the model will assess the level of trust between the buyer (as the trustor) and the sellers of the resources (as the trustees) (as the trust aim).
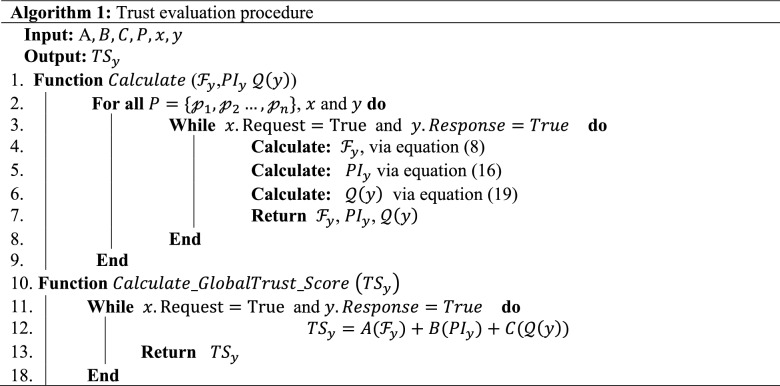


A trading task is defined here as the sale of a specified quantity of a resource product presented by the seller $y$ on the smart marketplace auction board to a buyer $x$ who has proven a desire to purchase such resource. The necessary trading task is event-based, geographical, time-sensitive, and almost real-time. Based on the proposed model, the following trust attributes are selected:

- Response Time: This attribute indicates how long it took for a seller $y$ to react to a buyer's demand. The response time (${\mathcal{R}}_{\mathcalligra{t}}$) of a product can be determine by difference between ${\mathcalligra{d}}_{\mathcalligra{t}}$ and ${\mathcalligra{r}}_{\mathcalligra{t}}$, where ${\mathcalligra{d}}_{\mathcalligra{t}}$ is the time of supply and ${\mathcalligra{r}}_{\mathcalligra{t}}$ is the time of demand. It is the amount of time that has passed between a buyer's demand for a resource and the seller's fulfillment of that demand. It is the difference in time between when a buyer demands a resource from a seller and when the seller successfully delivers the demanded resource. Therefore, response time can be computed as follows:
  2 $${\mathcal{R}}_{\mathcalligra{t}}=\frac{{\mathcalligra{d}}_{\mathcalligra{t}}-{\mathcalligra{r}}_{\mathcalligra{t}}}{{\mathcalligra{d}}_{\mathcalligra{t}}+{\mathcalligra{r}}_{\mathcalligra{t}}}$$
- Availability: This attribute plays a significant role in calculating a participant's trustworthiness. Prior to being broadcast on the sale panel (auction board), a resource must be available. Prior to allowing a seller $y$ to broadcast its bids on the sale panel, SG validates the presence of a resource product after evaluating the seller's id and the resource product ownership id, which it then verifies with RD. Therefore, the availability attribute indicates a seller's participation in social activities. As per this model, a resource product ${\mathcalligra{p}}_{y}$ is inaccessible even if a selling entity has advertised their offers but has not yet decided to trade them. Given that ${\mathcal{N}}_{adv}^{y}$ represent the frequency at which a given resource product was demanded to be advertised over a period and let ${\mathcal{N}}_{avl}^{y}$ represent the frequency at which the resource product was obtainable. Consequently, the availability of a resource is estimated using Eq. (3). Thus, once a buyer $x$ demands a particular resource product, then the scheme calculates the seller's resource product availability (${\mathcal{V}}_{{\mathcalligra{p}}_{y}}$).
  3 $${\mathcal{V}}_{{\mathcalligra{p}}_{y}}=\frac{{\mathcal{N}}_{avl}^{y}}{{\mathcal{N}}_{adv}^{y}}$$
- Success Rate: This attribute describes the number of successfully completed trading tasks for a given resource product ${\mathcalligra{p}}_{y}$. Let ${\mathcalligra{T}}_{{\mathcalligra{p}}_{y}}$ represent the number of profitable trades accomplished from ${\mathcal{N}}_{avl}^{x}$. The formula for calculating success rate is as follows:
  4 $$\eta =\frac{{\mathcalligra{T}}_{{\mathcalligra{p}}_{y}}}{{\mathcal{N}}_{avl}^{y}}$$
- Willingness: This attribute denotes the amount to which a seller $y$ collaborates with trading errands; therefore, a higher degree of willingness ${\mathcalligra{w}}$ indicates that the $y$ is more likely to be willing to complete a given trading task. Equation (5) can be used to calculate willingness as follow:
  5 $${\mathcalligra{w}}=\varphi \times \frac{{\mathcalligra{T}}_{{\mathcalligra{p}}_{y}}}{{\mu }_{{\mathcalligra{p}}_{y}}} .$$

Where ${\mu }_{{\mathcalligra{p}}_{y}}$ represents the number resource product requested, while $\varphi$ denotes the number of times the seller $y$ has engaged in resource product trade (trading task). It is computed using Eq. (6).

6 $$\varphi =\frac{{\mathcalligra{T}}_{{\mathcalligra{p}}_{y}}}{T},$$

where $T$ is time taken to complete the profitable trades.

- Integrity: This attribute reflects the degree to which the seller fulfills their commitments once a buyer expresses interest in purchasing a certain resource product. Once a resource product has been selected for a trading task, a seller $y$ with a high degree of integrity $\delta$ will not cancel the task for any reason. This attribute can be determined using the Eq. (7).
  7 $$\delta =1-\frac{{\mathcalligra{C}}_{{\mathcalligra{p}}_{y}}}{{\mathcalligra{T}}_{{\mathcalligra{p}}_{y}}},$$
  where ${\mathcalligra{C}}_{{\mathcalligra{p}}_{y}}$ is the trading tasks canceled from ${\mathcal{N}}_{avl}^{y}$.

Thus, from Eqs. 1, 2, 3, 4, 5, 6, the familiarity value ${\mathcal{F}}_{y}$ of a given seller can be computed as follow:

8 $${\mathcal{F}}_{y}=A\cdot {\mathcal{V}}_{{\mathcalligra{p}}_{y}}+\mathcalligra{a}\cdot \eta +\mathcalligra{b}\cdot {\mathcalligra{w}}+\mathcalligra{d}\cdot \delta +C\cdot {\mathcal{R}}_{\mathcalligra{t}}$$

where $\mathcalligra{a},\mathcalligra{b}and \mathcalligra{d}\in B.$ While *A, B, C* signifies the weights. Priority is given to a resource's availability rather than its response time: $A=0.5, B=0.3, C=0.1$.

### Personal interactions modelling

Personal interactions represent personal understandings and attitudes towards an object based on previous interactions. Using the present value, interaction results, and communication timestamps, a mathematical framework for personal interaction characteristics may be used to compute experiences. The personal interaction attribute model consists of an evaluation system for interaction results. Depending on the circumstances, several strategies may be used to forecast interaction outcomes. After each transaction, many smart marketplaces and reputation systems may employ implicit and explicit consumer feedback as their outputs. In certain reputation systems, an acknowledgement message creates a Boolean value, 0/1, to indicate a successful interaction.

Also required is a model for collecting personal interaction attributes. Cooperative contacts enhance the experience, whereas uncooperative contacts diminish it. Additionally, it decays without interacting^9,50^. Thus, there are three tendencies in personal interaction connection: Increase, Decrease, and Decay. These are evaluated based on interaction intensity, interaction values, and the value of their present experience. Consequently, a linear difference equation can describe trends in human interaction qualities. We provided a model of personal interaction attributes where the result of an encounter is either 0 (uncooperative) or 1 (cooperative). There are three tendencies in the model:

#### Increase in personal interaction (cooperative contact)

Using a linear differential equation, increase in personal interaction $\sigma$ at a time $\mathcalligra{t}$ can be expressed as follow:

9 $${\sigma }_{\mathcalligra{t}+1}={\sigma }_{\mathcalligra{t}}+ {\Delta \sigma }_{\mathcalligra{t}+1},$$

while,

10 $${\Delta \sigma }_{\mathcalligra{t}+1}=\tau -\frac{\tau }{{max}_{\sigma }}\times {\sigma }_{\mathcalligra{t}},$$

where ${\sigma }_{\mathcalligra{t}}$ is personal interaction at a time $\mathcalligra{t}$*,* and $\Delta {\sigma }_{\mathcalligra{t}}$ is the increase in the value of the personal interaction at a time $\mathcalligra{t}$. The highest increase value of the personal interaction is given as the parameter $\tau$, while the parameter ${max}_{\sigma }$ expresses the highest value of personal interaction, in most cases ${max}_{\sigma }>\tau$. For conveniency, we have adopted the same trust scale values ranging from 0 to 1 for the personal interaction attribute values such that, ${max}_{\sigma }=1$. Thus, Eq. (9) and (10) can be rewritten as follows:

11 $${\sigma }_{\mathcalligra{t}+1}={\sigma }_{\mathcalligra{t}}+\tau \times \left(1-{\sigma }_{\mathcalligra{t}}\right),$$

Or

12 $${\sigma }_{\mathcalligra{t}+1}=\left(1-\tau \right)\times {\sigma }_{\mathcalligra{t}}+\tau .$$

From Eq. (11), we can notice that the rise in personal interaction is quite great when the value of ${\sigma }_{\mathcalligra{t}}$ is small, but it approaches zero when the value of ${\sigma }_{\mathcalligra{t}}$ is close to 1.

#### Decrease in personal interaction (uncooperative contact)

The decrease in personal interaction can be expressed as follow:

13 $${\sigma }_{\mathcalligra{t}-1}=Max\langle {min}_{\sigma }, {\sigma }_{\mathcalligra{t}}-\alpha \times {\Delta \sigma }_{\mathcalligra{t}+1}\rangle ,$$

where $\alpha$ is a damping factor that controls the decline rate. Due to the fragile nature of the personal interaction relationship, the $\alpha$ parameter may be constant, or variable based on the circumstances, but it must always be greater than 1. ${min}_{\sigma }$ is a parameter that specifies the minimum value of the personal interaction (in this case, 0), ensuring that the value of the personal interaction cannot be lower.

#### Decay in personal interaction (no interaction)

When participants go for lengthy durations without interacting, they miss out on crucial human connection experience. However, the decrease rate may vary based on the present stage of the connection (i.e., the value of personal interaction). When the current state is low, suggesting a weak link between the two participants, a significant reduction happens, and when the status is high, a little reduction occurs. It is thus considered that frequent human engagement is necessary, yet strong bonds tend to persist even when cooperative interactions are not rewarded. Personal interactions with a high value are projected to deteriorate at a slower pace than those with a low value, since decay is inversely proportional to the interaction's present value. The theoretical model for the decay in the quality of interpersonal connections is as follows.

14 $${\mathcalligra{D}}_{\mathcalligra{t}-1}^{\sigma }=Max\langle {min}_{\sigma }, {\sigma }_{\mathcalligra{t}}-{\Delta \mathcalligra{D}}_{\mathcalligra{t}-1}\rangle ,$$

where

15 $${\Delta \mathcalligra{D}}_{\mathcalligra{t}-1}=\vartheta \times \left(1+\mu -\frac{{\sigma }_{\mathcalligra{t}-1}}{{max}_{\sigma }}\right),$$

where ${\mathcalligra{D}}_{\mathcalligra{t}-1}$ is the decay parameter, $\vartheta$ is a parameter specifying the least decay value of personal interaction that ensures even strong links will diminish if personal interaction is not maintained. $\mu$ is a parameter denoting the decay rate, which may be constant or variable depending on the circumstances.

Given the weights *A, B, C* applied to the three tendencies previously explained above (Eqs. 9, 10, 11, 12, 13, 14, 15), then the personal interaction ${PI}_{y}$ of buyer towards a trading task offered by a given seller can be expressed as:

16 $${PI}_{y}=A\cdot {\sigma }_{\mathcalligra{t}+1}+B\cdot {\sigma }_{\mathcalligra{t}-1}+C\cdot {\mathcalligra{D}}_{\mathcalligra{t}-1}^{\sigma }$$

As indicated by the personal interaction model, a high personal interaction value, such as a strong bond between a trustor and a trustee, can only be formed by a significant number of pleasant encounters in a short period of time. As time passes, it becomes more difficult to descend from a height. However, when the connection to the event is already tenuous, uncooperative encounters may inflict severe damage. The proposed personal interaction model has the potential to effectively transfer the experience link from the human social context to participants in the smart marketplace, as it is analogous to what occurs in the real human world.

### Public opinion modelling

In a smart marketplace based on SIoT, opinion is the consequence of interactions, and personal interaction is the sum of opinions from one participant to another. According to the personal interaction model proposed earlier, each buyer who has engaged with a certain seller has an opinion about that seller. And if all these participants express their opinions as recommendations about the seller to others, a model can be developed that aggregates these opinions to form the public opinion on the seller. Each opinion contributes uniquely to the public's opinion on the seller. The influence of a buyer's opinion on public perception of a seller depends on their personal interaction and the buyer's reputation. Therefore, effective public opinion models should include both personal interaction and public opinion on the buyer. A buyer with a high public opinion value contributes more to the public's perception of a seller than one with a low public opinion value.

This section discusses a method for determining public opinion values of trust for all participants in the blockchain-based smart marketplace network with a semi-centralized architecture. The procedure entails collecting information on the social ties of the buyer and seller. Nonetheless, this study included two significant obstacles: (a) varied weights of opinions from several participants to a certain participant; and (b) opinions might be both positive and negative. Positive opinions arise when personal interaction value leads to a rise in public opinion of the desired participant, while negative opinions should result in a decline in public perception value. Given $\gamma$ is the parameter that sets the threshold at which a particular personal interaction is evaluated as either positive or negative, then using the damping factor $\alpha$, both positive public opinion ${{\mathcal{Q}}}_{+ve}$ and negative public opinion ${{\mathcal{Q}}}_{-ve}$ can be expressed as follows:

17 $${{\mathcal{Q}}}_{+ve}\left(y\right)=\frac{1-\alpha }{n}+\alpha \times \left(\sum_{\forall x}{{\mathcal{Q}}}_{+ve}(x)\times \frac{\sigma \left(x,y\right)}{{\mathcalligra{r}}_{+ve}\left(x\right)}\right);\forall\,x\,that\,\sigma\,\left(x,n\right)>\gamma ,$$

18 $${{\mathcal{Q}}}_{-ve}\left(y\right)=\frac{1-\alpha }{n}+\alpha \times \left(\sum_{\forall x}{{\mathcal{Q}}}_{-ve}(x)\times \frac{\sigma \left(x,y\right)}{{\mathcalligra{r}}_{-ve}\left(x\right)}\right);\forall\,x\,that\,\sigma\,\left(x,n\right)<\gamma$$

19 $$\therefore {\mathcal{Q}}\left(y\right)=Max\left({min}_{{\mathcal{Q}}},{{\mathcal{Q}}}_{+ve}\left(y\right)-{{\mathcal{Q}}}_{-ve}\left(y\right)\right).$$

where $n$ is the total number of participants in the network, ${{\mathcal{Q}}}_{+ve}(x)$ and ${{\mathcal{Q}}}_{-ve}(x)$ are the positive and negative public opinion of the participant $x$ respectively, while ${\mathcal{Q}}\left(y\right)$ is the public opinion on the participant $y$ (seller). The parameter ${\mathcalligra{r}}_{+ve}\left(x\right)={\sum }_{\sigma \left(x,y\right)>\gamma }\sigma \left(x,y\right)$ is the summation of all positive public opinion values that a participant $x$ (buyer) is currently sharing, while ${\mathcalligra{r}}_{-ve}\left(x\right)={\sum }_{\sigma \left(x,y\right)<\gamma }\sigma \left(x,y\right)$ is the summation of all the negative ones. The parameter ${min}_{{\mathcal{Q}}}$ is the minimal value of public opinion (i.e., 0) which ensures that the public opinion value will not fall beyond it. The damping factor $\alpha$ which describes the rate at which the decay occurs, here is chosen to be 0.85 as suggested by many studies such as^9,16^.

After implementing a sequence of iterations all through the network, Eqs. 17, 18, 19) generate a normalized probability distribution of the public opinions (positive, negative, and overall public opinions), as well as determining and updating public opinion values for all participants in the smart marketplace network after each iteration. By combining categorization machine learning techniques with a semi-centralized blockchain architecture, the whole smart marketplace network can be subdivided into smaller subgroups, making the proposed public opinion model feasible to implement.

### Resource product selection

After successfully calculating sellers' familiarity value $({\mathcal{F}}_{y})$, personal interaction value $({PI}_{y})$, public opinion value $({\mathcal{Q}}\left(y\right))$, and global trust value, the resource product is chosen. This can be accomplished by:

20 $${P}_{select}^{y}={arg}_{{\mathcalligra{p}}_{y}} max {TS}_{y}\left(x,y\right)$$

$$s.t\left\{\begin{array}{c}{\mathcal{F}}_{y}\ge {T}_{{\mathcal{F}}_{y}},\\ {PI}_{y}\ge {T}_{{PI}_{y}},\\ Q\left(y\right)\ge {T}_{{\mathcal{Q}}\left(y\right)}.\end{array}\right.$$

${T}_{{\mathcal{F}}_{y}}$, ${T}_{{PI}_{y}}$, and ${T}_{{\mathcal{Q}}\left(y\right)}$ represent the levels of sellers' familiarity, personal interaction, and public opinion, respectively. The thresholds aid buyers in selecting the most qualified seller. Additionally, the criteria prevent bad actors from feigning trust in their actions. There is a possibility that prospective sellers will use deceptive means to gain the object's trust by exaggerating their abilities and promising to provide the requested resource product. Even if the seller has an overall high trust value, they are still being dishonest with their buyers if their public opinion rate is below the required minimum^9^.

## Security analysis

This section identifies and discusses six distinct trust-related threats based on the proposed adversary model. Similarly, we examined security analysis to determine how well the proposed MarketTrust model resists the attacks.

**Opportunistic service attack** In this paper, we assume that a decrease in a seller's trustworthiness corresponds to a decrease in the likelihood that a trading task will be completed. If a seller has a poor reputation, the likelihood of selecting a resource product from the malicious seller is reduced. As a result, it will be unable to rapidly enhance its credibility by offering an abundance of resource products. Even more so, poor performance will be severely punished, leading to longer and more substantial negative effects on evaluation that cannot be immediately mitigated by an opportunistic service attack.

**Whitewashing attack** In this kind of attack, an adversarial seller attempts to reframe the scheme's reliability by modifying or introducing a fresh identifier. As all participants in the Ethereum blockchain will be notified of any modifications to the blockchain via event logs, using the unique 20-byte EAs issued to each user of the network is one way to circumvent this issue. However, before the modification can be implemented, all network participants must agree to it. Therefore, even if an adversary tries to modify or replace an EA or alter the event logs, it will be detected due to the fact that all produced events are tamper-resistant and certified by the smart contract, securing it against such threats. Similarly, repairing trustworthy social links involves time and money, and a malicious seller must reject all trust that specific buyers have already developed.

**Self-promoting attack** The attack is thwarted because the buyer will not accept the potentially malicious seller's own recommendation, as determined by the model using the trading task histories of both parties. The dishonest seller fabricating widespread support in the form of positive public opinions is a novel form of attack. But in this trust model, this strategy is similarly ineffective, as these fabricated public opinions will carry little weight in the eyes of the buyers, and the trust-related information they contribute will be irrelevant to the evaluation of the trustworthiness of the malicious sellers.

**Defamation attacks** The less impact the negative buyer's opinion had on the model's evaluation of a seller, the more favorable public opinion a buyer received. Therefore, for a malicious buyer to conduct such an attack against a reputable seller, the malevolent buyer have to initially create a strong communal connection with the seller and must have as little access as possible to trust-related public opinions obtained from other sources regarding the seller's trustworthiness. It will be difficult for defamation attacks to succeed because malicious buyers will have difficulty estimating or erroneously influencing public opinion on grounds of trust.

**Voting attacks** The requirements for a voting attack are comparable to those for a defamation attack, but more difficult to fulfill. Consequently, the attacker must guarantee that the requirements ${\mathcal{F}}_{y}\ge {T}_{{\mathcal{F}}_{y}}$, ${PI}_{y}\ge {T}_{{PI}_{y}}$ and ${\mathcal{Q}}\left(y\right)\ge {T}_{{\mathcal{Q}}\left(y\right)}$ are satisfied, as given in the Eq. (20), in addition to the constraints against defamation attacks described above. This condition is, however, very difficult for the attacker to satisfy. This makes it more difficult for an adversary to conduct a voting attack.

**Discriminatory attack** In this strategy, the adversary lacks the social connections necessary for the calculation of trust integration from multiple sources. An attacker must first identify as many social ties between options as possible to generate dispute, which is a time-consuming and unrealistic task. It is also risky to launch a discriminatory attack, as the attacker can be penalized for mistrust. In addition, a discriminatory attack on a prominent buyer may have a substantial effect on the attacker's trustworthiness. Accordingly, discrimination-based attacks are ineffective under this trust paradigm.

## Implementation and performance analysis

In this section, we will discuss the experimental settings and performance criteria applied to evaluate the proposed MarketTrust model. Additionally, the experimental findings were compared to those obtained using the reference models established by Fortino et al.^42^ and M. Kamran et al.^44^. We selected the reference models based on their closeness to the present and contextual relevance to our own models.

### Simulation setup

For the studies, a PC with an Intel(R) Core (TM) i7-6700 CPU @ 3.40 GHz 3.41 GHz was employed. The MarketTrust implementation was performed using Solidity language and python modules. During the implementation and testing, the Metamask Ethereum wallet, the Rinkeby testnet, and a Proof-of-Authority (PoA) consensus mechanism were used. To eliminate prejudice and get the most accurate observations and results, the models were applied in a similar environmental setup. In addition, the following model parameters were used throughout simulations:

- Each trade circle interaction scenario comprised of a randomly selected seller and buyer, and by extending the population in each scenario, the smart marketplace environment population was capped at 500 participants (sellers and buyers).
- Interactions in each trade circle occurred in minutes. Thus, in one thousand minutes, one thousand interactions took place, where every member functioned as either a seller or a buyer in distinct trade circles.
- In different trading circles, the percentage of dishonest sellers ranged from 10 to 100%.
- The seller's attributes thresholds for familiarity ${T}_{{\mathcal{F}}_{y}}$, personal interaction ${T}_{{PI}_{y}}$, and public opinion ${T}_{{\mathcal{Q}}\left(y\right)}$ for resource product selection values were initialized as 0.60, 0.70, and 0.60, respectively.

Notably, we were able to track the development of each task thanks to the smooth running of events that generated sufficient data. The proposed code for the MarketTrust smart contract was tested in a number of different scenarios to verify its logic. The safety of the smart contract was analyzed using the Oyente tool^51^. According to the results, the smart contract has 68% EVM Code Coverage. Similarly, the results showed that the code does not contain any of the well-known security issues, such as reentrancy, timestamp dependence, transaction dependency, or a parity multisig problem.

### Performance evaluations

Several criteria, including average trust scores, average execution delay, computational cost per gas use, and our model's resistance towards considered threats, were utilized to evaluate the performance of MarketTrust. The outcomes of the assessment are detailed in the sections that follow.

#### Evaluation of trust and system performance

**Figure 2:**
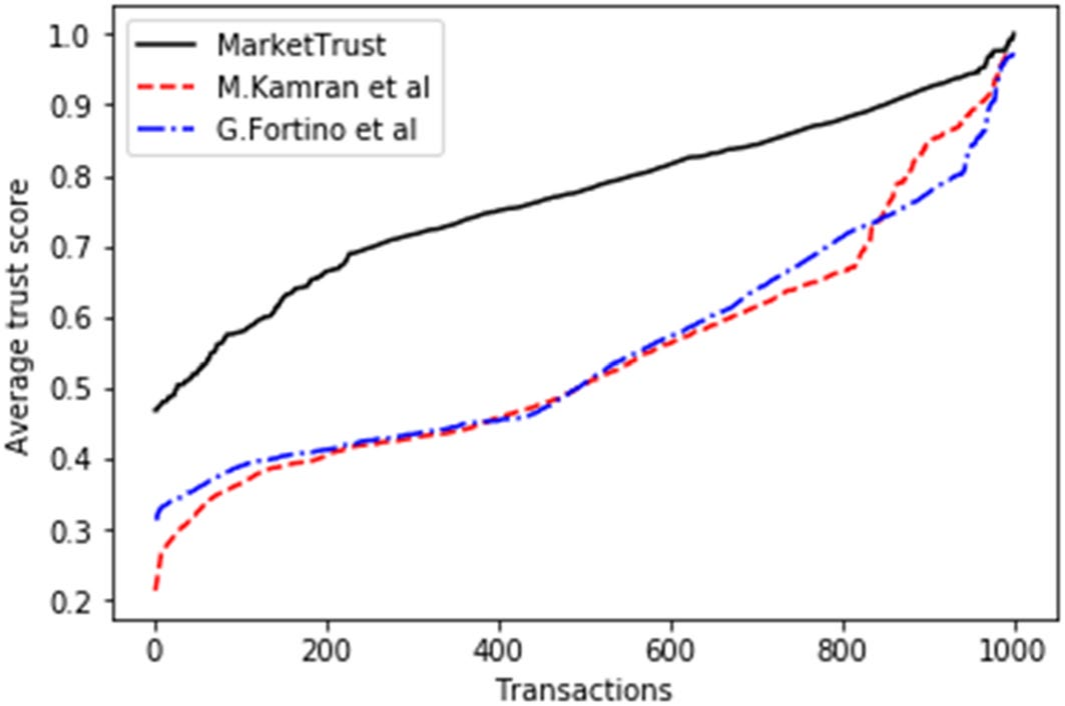
Average trust score.

Figure 2 illustrates the results in terms of average trust score for all models. An average trust score is the overall measurement of reviewer satisfaction, based on the parameters we have established, which are "familiarity," "personal interactions," and "public perception in the midst of fraudulent sellers. The trust score depicts the level of confidence in a particular trading session. It is determined by the relationship between the current session's values and behavior and the seller's past. In addition, it determines whether the session poses a risk relative to the general population.

In comparison to both benchmark models, simulation results indicate that the MarketTrust model achieved a 21.99% higher trust score. By integrating "familiarity," "personal interactions," and "public perception," the proposed model is capable of generating a realistic trust score in the mist of fraudulent sellers. On the other hand, benchmark models are unable of computing an accurate trust score for sellers owing to the probable influence of fraudulent sellers on the feedbacks gathered from system participants. As a consequence, the benchmark models ineffectively calculate the trust ratings of the sellers. Moreover, the findings demonstrate that, even when the prospective buyer have no substantial number of personal interactions over a long time, it can still make an accurate assessment using the available public opinion outputs regarding the prospective seller. This feature allows potential buyers to distinguish between fraudulent and legitimate sellers.

**Figure 3:**
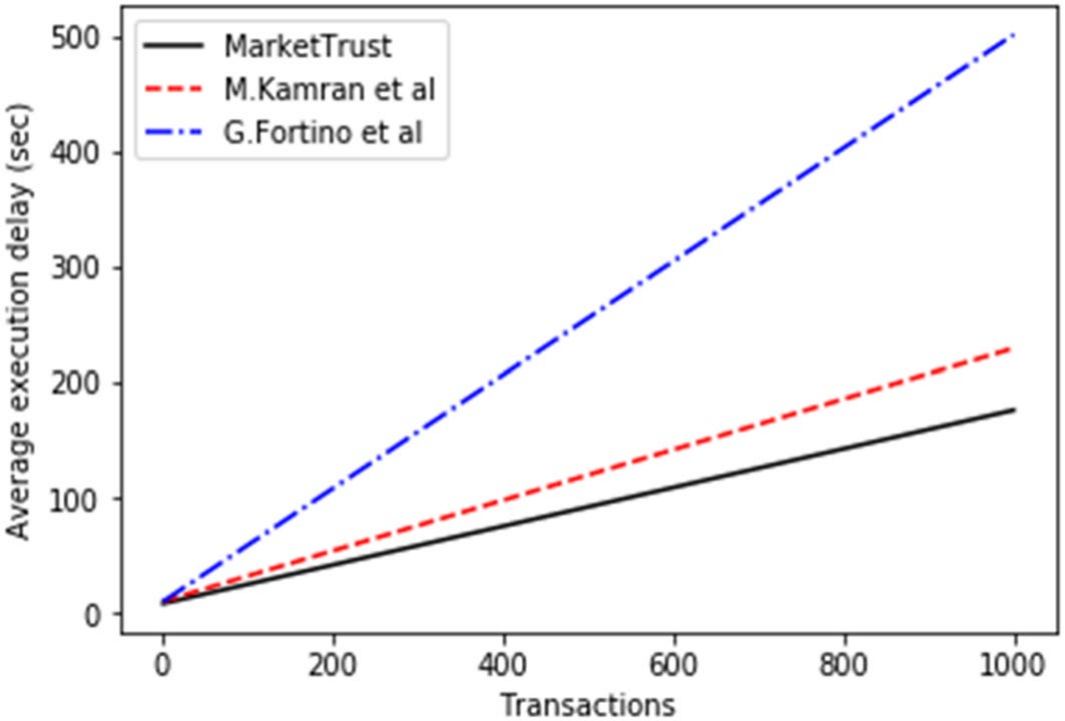
Average execution delay.

**Figure 4:**
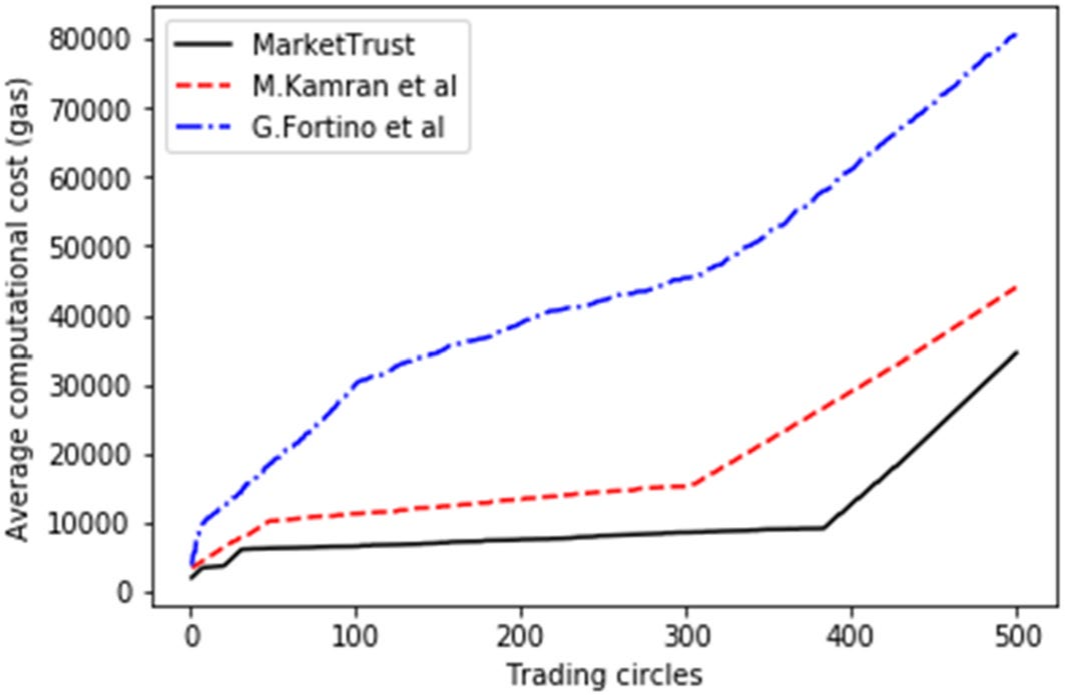
Average computational cost.

This model relates the execution delay to the average transaction block confirmation time (block generation time). While computing costs are proportional to gas usage per transaction unit. Figures 3 and 4 depict simulation results indicating that our model had a reduced average execution delay and computational cost than the benchmark models. This is because processing-intensive clustering approaches have been eliminated. In addition, chosen nodes maintain the PoA consensus method used by the proposed model. While the proof-of-work (POW) consensus process used by other models necessitates miners to add transactions to the chain. Consequently, our model recorded 47.698 percent shorter average delay for 500 parties than the benchmark models.

#### Evaluation of system resiliency

Several trade cycles that occur between trading partners were pre-programmed during the first phase so that the prospective buyer could obtain an early grasp of the familiarity, personal interactions, and public opinion properties. To properly evaluate the resilience of all models, the proportion of malicious sellers impacting the system throughout the trading cycle was varied from 10 to 100%. In this research, resilience is defined as the ratio between the model's resistance to a specific attack (among the attacks under consideration in this model) and the proportion of dishonest sellers undertaking the same attack within the system. Initial resilience values for all models were programmed to be one unit (1 unit), after which all interaction processes are allowed to continue until one thousand transactions are recoded in each proportion.

**Figure 5:**
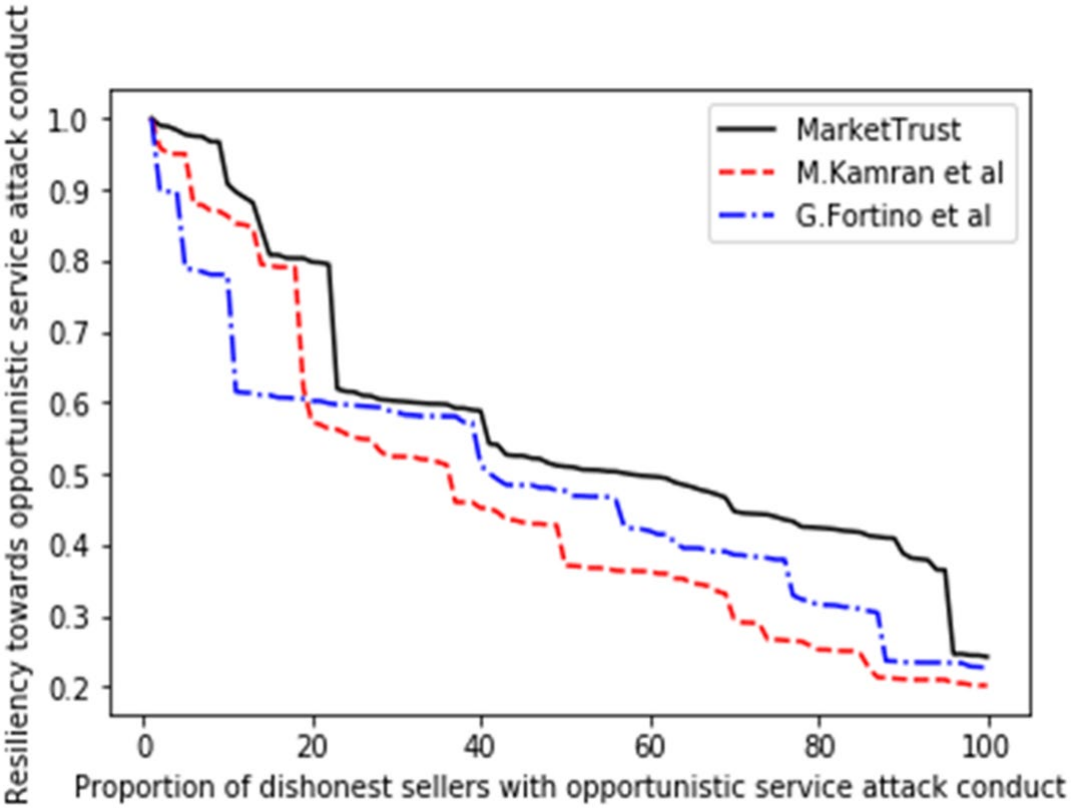
Resilience on opportunistic service attack.

**Figure 6:**
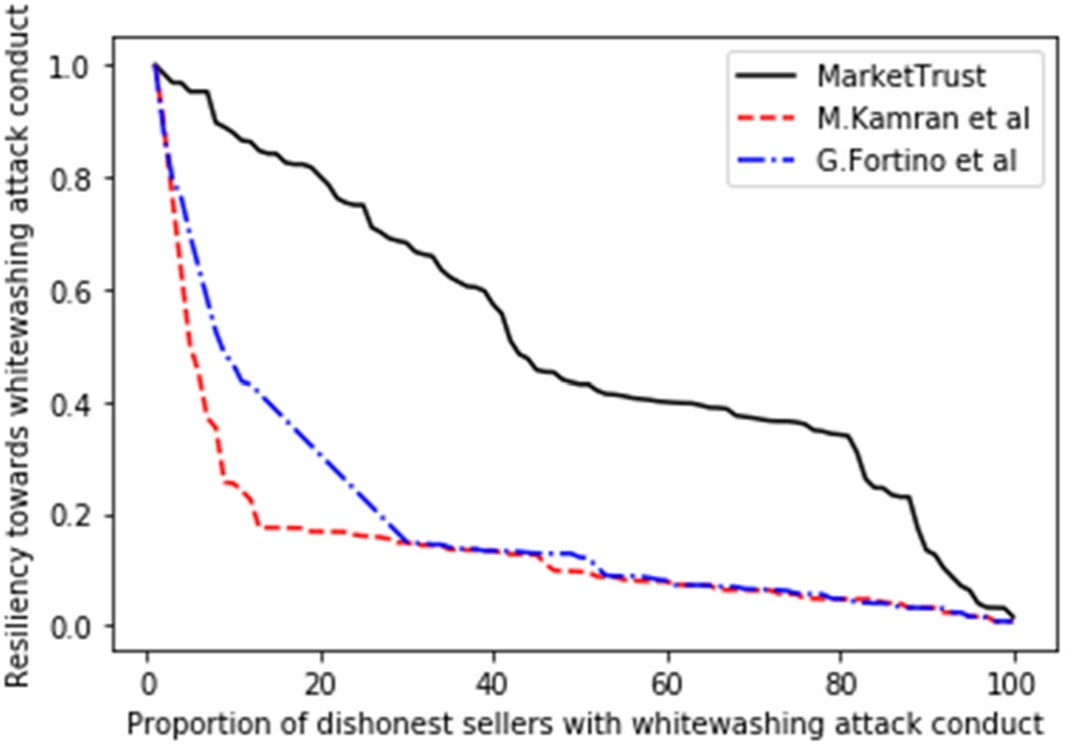
Resilience on whitewashing attack.

**Figure 7:**
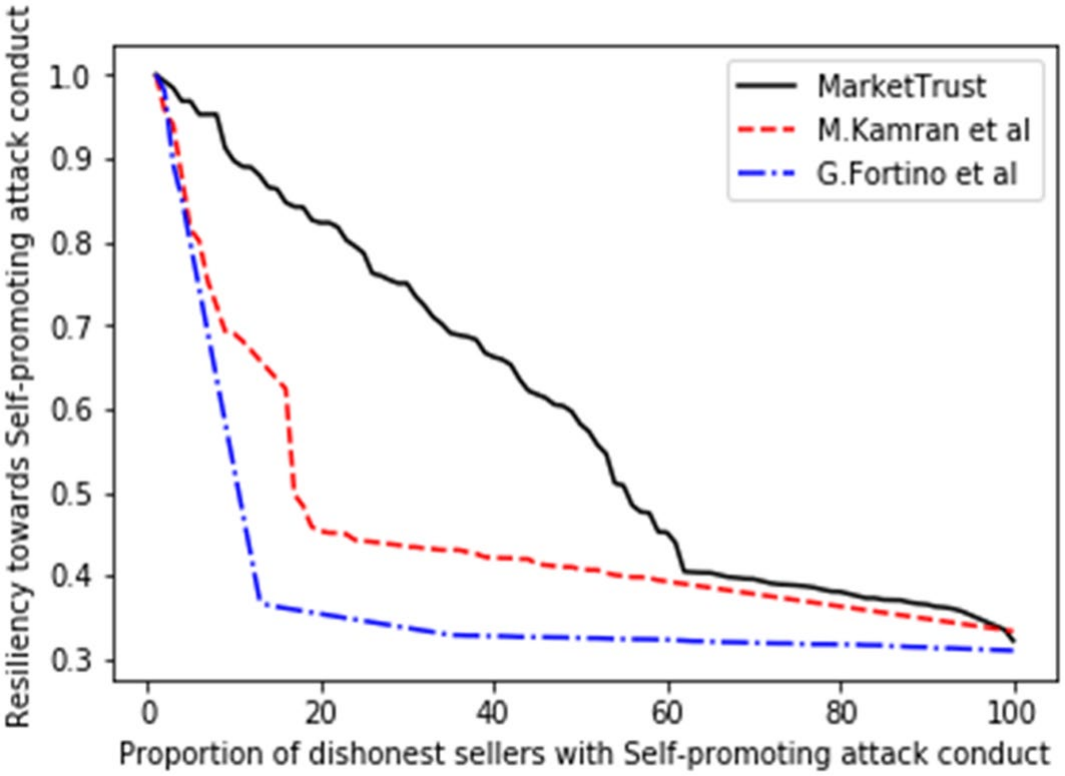
Resilience on self-promotion attack.

**Figure 8:**
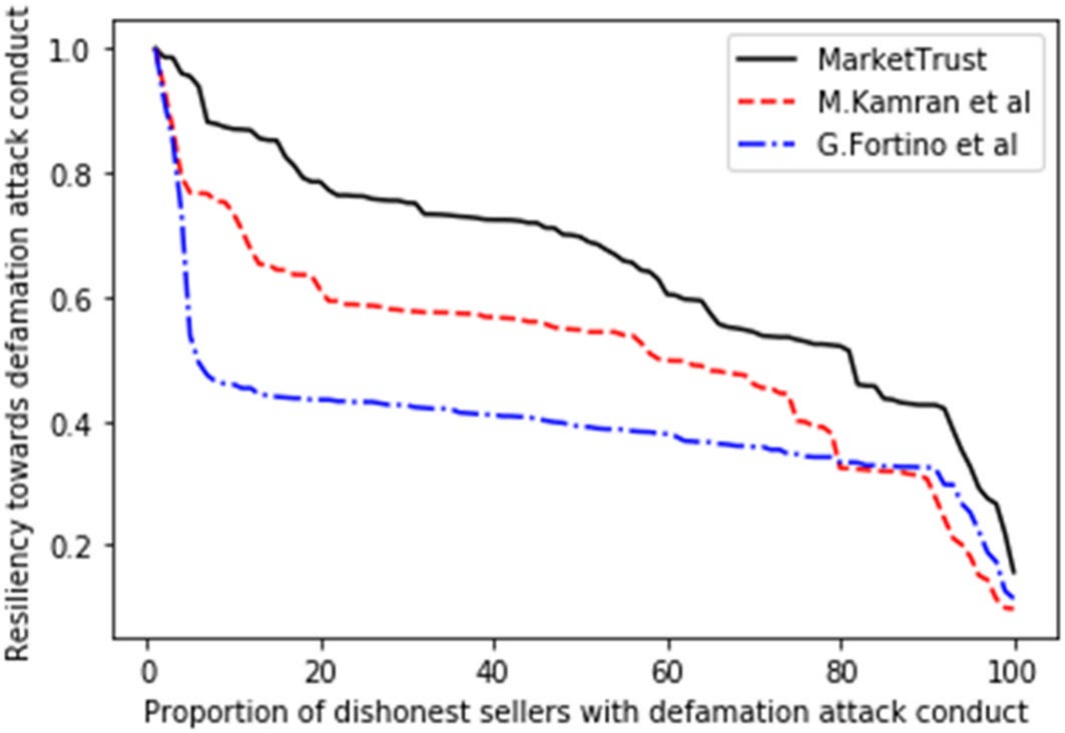
Resilience on defamation attack.

**Figure 9:**
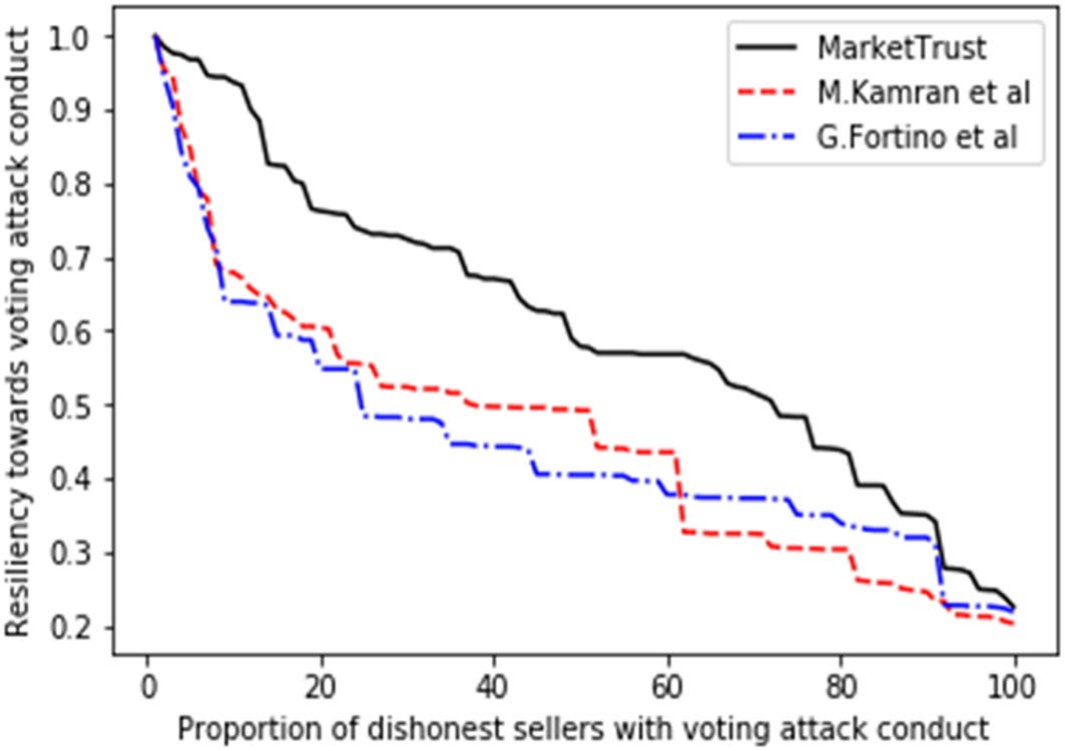
Resilience on voting attack.

**Figure 10:**
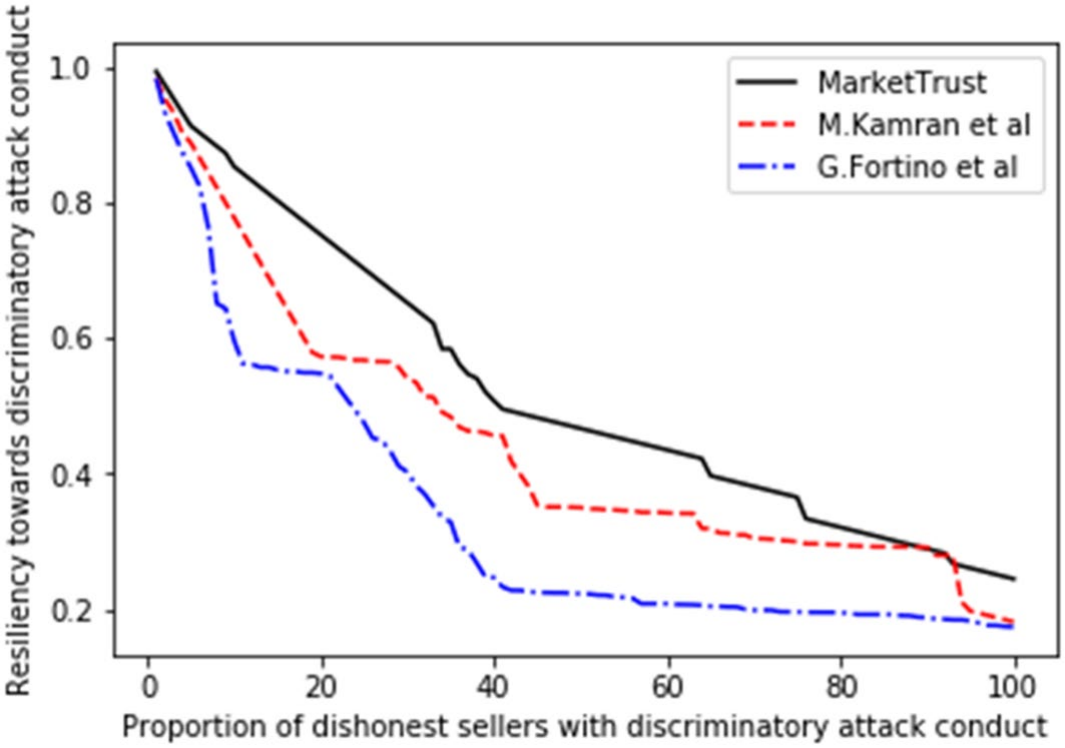
Resilience on discrimatory attack.

Consequently, the performance of our proposed model was measured in comparison to the existing models of G. Fortino et al.^42^ and M. Kamran et al.^44^, with the findings shown in Figs. 5, 6, 7, 8, 9, 10. Compared to other attacks, it is evident that the discriminatory service attack was reported to be the one with most negative impact on the proposed model. Notwithstanding, the findings reveal that the proposed model has a greater tendency for endurance than the reference models. Consequently, this indicates the confidence with which the proposed model can aid a prospective buyer in identifying a trustworthy seller, even amid malicious sellers.

### Comparative analysis

From the findings shown in Figs. 2, 3, 4, 5, 6, 7, 8, 9, 10, we summarize and compare the primary security aspects as well as other critical features related with the resilience of the models in Table 3. According to the summary, the approach given by G. Fortino et al. which was based on multi-agent systems satisfies fewer security requirements and is thus susceptible to many trust-based attacks. Relatively, the M. Kamran et al. scheme surpasses the G. Fortino et al. scheme, which employs a similar multi-agent systems blockchain trusted suite technique to concurrently accomplish three goals, namely trust, collaboration, and secrecy.

**Table 3:** Comparison of security parameters.

Security parameters	Kamran et al.^44^	G. Fortino et al.^42^	MarketTrust
Resistance to opportunistic service attack	Yes	Yes	Yes
Resistance to whitewashing attack	No	No	Yes
Resistance to self-promoting attack	No	No	Yes
Resistance to defamation attacks	Yes	Yes	Yes
Resistance to voting attacks	Yes	Yes	Yes
Resistance to discriminatory attack	Yes	No	Yes
Secured trust computation process	No	No	Yes
High scalability	No	No	Yes

Both models use clustering (grouping/multi-agent) methodologies, which often result in unsafe double-spending and need considerable computing resources. Thus, the systems were based on predications of their participants profiles similarities, which does not ensure consistency and creates difficulties for associations in terms of cluster suggestion. Consequently, the models are susceptible to several security concerns, including whitewashing attacks, self-promotion, and discriminating threats, consequential to skewed trust calculation.

The reference schemes were designed with a limited number of individuals in view. Therefore, as the participants increases in numbers, the effect of the schemes diminishes. Nevertheless, the proposed MarketTrust model is scalable and resistant to all security concerns evaluated in this work and can therefore be used in dynamic settings.

## Conclusion

In this paper, we present a decentralized trust management solution for smart marketplaces based on Social IoT. This approach permits the measurement of trust based on social links between trade parties. Individuals (buyers) commonly base their appraisal of a seller's trustworthiness on three key pillars: familiarity with the seller (Familiarity), prior encounters with the seller (personal interactions), and public impression of the seller (public perception). The approach employs a resource product selection method based on sellers' degrees of familiarity, personal interaction, and public opinion. This helps buyers choose the most qualified seller and prevents bad actors from pretending to be trustworthy. Utilizing blockchain technology, the methodology ensures confidentiality and anonymity while providing a high degree of trust computation accuracy.

Experimental simulation was used to evaluate the performance of the proposed model; the results indicate that the proposed framework can effectively evaluate the trustworthiness of trading entities and outperforms other relevant existing models in terms of achieving high trust scores, low execution delay, and cost-efficiency. Additionally, we proved that the proposed model is impervious to typical threats on Social IoT trust management systems.

Some fundamental limitations of the proposed model include limited artificial data sources and a reduced sample size for population scenarios based on simulations of smart marketplaces. However, the future work will include incorporating artificial intelligence into the framework to strengthen the process for evaluating trust in Social IoT utilizing varied data sources, such as network data and behavior data. In addition, we intend to simulate increasingly complicated smart marketplace scenarios and test the methodology using real-world implementations.

## Data Availability

The datasets generated and/or analyzed during the current study are available from the corresponding author on reasonable request.

## References

[1] Obaidat MA, Obeidat S, Holst J, Al Hayajneh A, Brown J (2020). A comprehensive and systematic survey on the internet of things: Security and privacy challenges, security frameworks, enabling technologies, threats, vulnerabilities and countermeasures. Computers.

[2] Truong NB, Lee H, Askwith B, Lee GM (2017). Toward a trust evaluation mechanism in the social internet of things. Sensors.

[3] Farhadi B, Masoud Rahmani A, Asghari P, Hosseinzadeh M (2021). Friendship selection and management in social internet of things: A systematic review. Comput. Netw..

[4] LafiAljohani S, Alenazi MJF (2020). Evaluation of WSN’s resilience to challenges in smart cities. Int. J. Comput. Commun. Eng..

[5] Zhou Y, Yu FR, Chen J, Kuo Y (2020). Cyber-physical-social systems: A state-of-the-art survey, challenges and opportunities. IEEE Commun. Surv. Tutorials.

[6] Landaluce H, Arjona L, Perallos A, Falcone F, Angulo I, Muralter F (2020). A review of iot sensing applications and challenges using RFID and wireless sensor networks. Sensors.

[7] Malekshahi Rad M, Rahmani AM, Sahafi A, Nasih Qader N (2020). Social Internet of Things: vision, challenges, and trends. Hum.-Centric Comput. Inf. Sci..

[8] Sharma V, You I, Andersson K, Palmieri F, Rehmani MH, Lim J (2020). Security, privacy and trust for smart mobile-Internet of Things (M-IoT): A survey. IEEE Access.

[9] Latif R (2022). ConTrust: A Novel Context-Dependent Trust Management Model in Social Internet of Things. IEEE Access.

[10] Aparicio M, Costa CJ, Moises R (2021). Gamification and reputation: Key determinants of e-commerce usage and repurchase intention. Heliyon.

[11] Rajendran S, Jebakumar R (2022). Friendliness based trustworthy relationship management (F-TRM) in social internet of things. Wirel. Pers. Commun..

[12] Altaf A, Abbas H, Iqbal F, Khan FA, Rubab S, Derhab A (2021). Context-oriented trust computation model for industrial Internet of Things. Comput. Electr. Eng..

[13] Al-khafajiy M (2020). COMITMENT: A fog computing trust management approach. J. Parallel Distrib. Comput..

[14] Abou-Nassar EM, Iliyasu AM, El-Kafrawy PM, Song OY, Bashir AK, El-Latif AAA (2020). DITrust chain: Towards blockchain-based trust models for sustainable healthcare IoT systems. IEEE Access.

[15] Chen IR, Guo J, Bao F (2016). Trust management for SOA-based IoT and its application to service composition. IEEE Trans. Serv. Comput..

[16] Khani, M., Wang, Y., Orgun, M. A., & Zhu, F. Context-aware trustworthy service evaluation in social internet of things. In *Lecture Notes in Computer Science (including subseries Lecture Notes in Artificial Intelligence and Lecture Notes in Bioinformatics)* (2018).

[17] Yu B, Wright J, Nepal S, Zhu L, Liu J, Ranjan R (2018). Trust chain: Establishing trust in the iot-based applications ecosystem using blockchain. IEEE Cloud Comput..

[18] Aslam MJ, Din S, Rodrigues JJPC, Ahmad A, Choi GS (2020). Defining service-oriented trust assessment for social internet of things. IEEE Access.

[19] Farahbakhsh B, Fanian A, Manshaei MH (2021). TGSM: Towards trustworthy group-based service management for social IoT. Internet of Things.

[20] Wu H, Dudder B, Wang L, Sun S, Xue G (2022). Blockchain-based reliable and privacy-aware crowdsourcing with truth and fairness assurance. IEEE Internet Things J..

[21] Latif S, Idrees Z, Ahmad J, Zheng L, Zou Z (2021). A blockchain-based architecture for secure and trustworthy operations in the industrial Internet of Things. J. Ind. Inf. Integr..

[22] Uddin MA, Stranieri A, Gondal I, Balasubramanian V (2021). A survey on the adoption of blockchain in IoT: Challenges and solutions. Blockchain Res. Appl..

[23] Yakubu BM, Khan MI, Javaid N, Khan A (2021). Blockchain-based secure multi-resource trading model for smart marketplace. Computing.

[24] Waleed M, Latif R, Yakubu BM, Khan MI, Latif S (2021). T-smart: Trust model for blockchain based smart marketplace. J. Theor. Appl. Electron. Commer. Res..

[25] Abdelghani, W., Zayani, C. A., Amous, I., & Sèdes, F. Trust management in social internet of things: A survey. in *Lecture Notes in Computer Science (including subseries Lecture Notes in Artificial Intelligence and Lecture Notes in Bioinformatics)* (2016).

[26] Al Muhtadi J, Alamri RA, Khan FA, Saleem K (2021). Subjective logic-based trust model for fog computing. Comput. Commun..

[27] Wylde V (2022). Cybersecurity, data privacy and blockchain: A review. SN Comput. Sci..

[28] Fleischer A, Ert E, Bar-Nahum Z (2022). The role of trust indicators in a digital platform: A differentiated goods approach in an Airbnb market. J. Travel Res..

[29] Chen L, Rashidin MS, Song F, Wang Y, Javed S, Wang J (2021). Determinants of consumer’s purchase intention on fresh E-commerce platform: Perspective of UTAUT model. SAGE Open.

[30] Rodgers W, Guiral A, Gonzalo JA (2019). Trusting/distrusting auditors’ opinions. Sustain.

[31] Yang Z, Yang K, Lei L, Zheng K, Leung VCM (2019). Blockchain-based decentralized trust management in vehicular networks. IEEE Internet Things J..

[32] Ali, S., Javaid, N., Javeed, D., Ahmad, I., Ali, A., & Badamasi, U. M. (2020) A blockchain-based secure data storage and trading model for wireless sensor networks. In *Advances in Intelligent Systems and Computing* (2020).

[33] Firdaus M, Rahmadika S, Rhee KH (2021). Decentralized trusted data sharing management on internet of vehicle edge computing (Iovec) networks using consortium blockchain. Sensors.

[34] Wang Y, Chen P, Wu B, Wan C, Yang Z (2022). A trustable architecture over blockchain to facilitate maritime administration for MASS systems. Reliab. Eng. Syst. Saf..

[35] Hasan HR, Salah K, Jayaraman R, Yaqoob I, Omar M (2021). Blockchain architectures for physical internet: A vision, features, requirements, and applications. IEEE Netw..

[36] Abunadi I (2022). Federated learning with blockchain assisted image classification for clustered UAV networks. Comput. Mater. Contin..

[37] Johar S, Ahmad N, Durrani A, Ali G (2021). Proof of pseudonym: Blockchain-based privacy preserving protocol for intelligent transport system. IEEE Access.

[38] Kim TH (2019). A novel trust evaluation process for secure localization using a decentralized blockchain in wireless sensor networks. IEEE Access.

[39] Kochovski P, Gec S, Stankovski V, Bajec M, Drobintsev PD (2019). Trust management in a blockchain based fog computing platform with trustless smart oracles. Fut. Gen. Comput. Syst..

[40] Shala B, Trick U, Lehmann A, Ghita B, Shiaeles S (2020). Blockchain and trust for secure, end-user-based and decentralized IoT service provision. IEEE Access.

[41] Weerapanpisit P, Trilles S, Huerta J, Painho M (2022). A decentralised location-based reputation management system in the IoT using blockchain. IEEE Internet Things J..

[42] Fortino G, Messina F, Rosaci D, Sarné GML (2020). Using blockchain in a reputation-based model for grouping agents in the Internet of Things. IEEE Trans. Eng. Manag..

[43] Fortino G, Messina F, Rosaci D, Sarné GML (2018). Using trust and local reputation for group formation in the Cloud of Things. Futur. Gener. Comput. Syst..

[44] Kamran M, Rasheed MB (2022). A blockchain-enabled trust aware energy trading framework using games theory and multi-agent system in smat grid. SSRN Electron. J..

[45] Wei, L., Wu, J., & Long, C. (2020) Enhancing Trust Management via Blockchain in Social Internet of Things. In *Proceedings—2020 Chinese Automation Congress, CAC 2020* (2020)

[46] Akbar NA, Muneer A, Elhakim N, Fati SM (2021). Distributed hybrid double-spending attack prevention mechanism for proof-of-work and proof-of-stake blockchain consensuses. Fut. Internet.

[47] Iftikhar Z (2021). Privacy preservation in resource-constrained iot devices using blockchain—A survey. Electronics.

[48] Ahmed AIA, Ab Hamid SH, Gani A, Khan S, Khan MK (2019). Trust and reputation for Internet of Things: Fundamentals, taxonomy, and open research challenges. J. Network Comput. Appl..

[49] Zarour M (2020). Evaluating the impact of blockchain models for secure and trustworthy electronic healthcare records. IEEE Access.

[50] Wei L, Wu J, Long C, Li B (2021). On designing context-aware trust model and service delegation for social Internet of Things. IEEE Internet Things J..

[51] Luu, L., Chu, D. H., Olickel, H., Saxena, P., & Hobor, A. Making smart contracts smarter. In *Proceedings of the ACM Conference on Computer and Communications Security*, 2016, pp. 254–269 (2016).

